# A multidisciplinary approach to treatment for osteochondroma of the mandibular condyle and oral rehabilitation: A case report

**DOI:** 10.1097/MD.0000000000044359

**Published:** 2025-09-12

**Authors:** Hyun Nam, Da Eun Ko, Kyung Su Shin

**Affiliations:** a Department of Orofacial Pain and Oral Medicine, Armed Forces Capital Hospital, Seongnam, South Korea; b Dental Implant Clinic, Armed Forces Capital Hospital, Seongnam, South Korea; c Department of Oral and Maxillofacial Pathology, Wonkwang University Daejeon Dental Hospital, Daejeon, South Korea; d Department of Oral and Maxillofacial Surgery, Armed Forces Capital Hospital, Seongnam, South Korea.

**Keywords:** dental implant, multidisciplinary therapy, oral rehabilitation, osteochondroma, temporomandibular joint

## Abstract

**Rationale:**

Osteochondromas are rare benign tumors that can occur in the temporomandibular joint (TMJ). These lesions can cause functional and aesthetic impairments of the jaw. Most studies have discussed surgical management and reconstruction within a single department. Establishing a patient-specific treatment plan through a multidisciplinary approach, involving various dental specialists can lead to better clinical outcomes. This case report illustrates the successful multidisciplinary management of a TMJ osteochondroma, emphasizing the importance of comprehensive diagnostic imaging, surgical intervention, and coordinated postoperative care.

**Patient concerns:**

A 50-year-old male visited the hospital to consult about implant treatment for the missing 1st and 2nd molar region in his right mandible. Imaging studies revealed a pedunculated mass on the right condyle.

**Diagnoses:**

Based on clinical and radiographic examinations, a provisional diagnosis of osteochondroma was established. After the surgery, the final diagnosis was confirmed by an oral and maxillofacial pathologist as osteochondroma and secondary synovial chondromatosis of the right TMJ.

**Interventions:**

A multidisciplinary team discussed the patient’s treatment plan. The patient underwent a low condylectomy and dental implant surgery followed by rehabilitation comprising physical therapy, jaw exercises, and the use of an occlusal stabilization splint.

**Outcomes:**

Postoperative evaluations showed gradual improvement in jaw function and stable occlusion. Six months after surgery, the patient had adapted well to the new occlusion and expressed satisfaction with the rehabilitation outcome, with follow-up imaging confirming the absence of recurrence.

**Lessons:**

Multidisciplinary management is crucial for achieving favorable outcomes in the treatment of TMJ osteochondromas. This case underscores the importance of comprehensive and coordinated treatment strategies.

## 1. Introduction

Osteochondromas are benign, cartilage-capped, exophytic lesions that typically arise from bones. Osteochondroma is the most common benign bone tumor, accounting for approximately 35% to 50% of benign bone tumors and 8% to 15% of all bone tumors overall.^[1]^ Although they most commonly occur in the long bones, they can also develop in the craniofacial region, including the temporomandibular joint (TMJ).^[2]^ TMJ osteochondromas are rare, with an incidence of <1% of all osteochondromas. Among craniofacial cases, the mandibular condyle is the most frequently affected site, accounting for up to 88.3% of jawbone osteochondromas.^[1,2]^ Although often painless, these lesions can cause mandibular deviation, open bite, and facial asymmetry.^[3]^

Histologically, osteochondromas consist of a cartilage-capped bony projection that is continuous with the underlying cortical and medullary bone, characterized by endochondral ossification and organized chondrocytes resembling epiphyseal cartilage.^[4]^

Surgical excision remains the primary treatment modality for TMJ osteochondroma.^[5–8]^ The surgical management varies depending on lesion size and involvement.^[5]^ Local excision is suitable for small lesions, while low condylectomy is commonly performed for larger tumors.^[6]^ In severe cases with condylar deformity or facial asymmetry, total condylectomy with joint reconstruction or combined orthognathic surgery may be required.^[7,8]^

A recent systematic review reported excellent surgical outcomes for TMJ osteochondroma, with a low recurrence rate of 0.22% and functional improvements.^[9]^ Importantly, multidisciplinary management, including adjunctive procedures such as orthognathic surgery and occlusal rehabilitation, plays a key role in optimizing both functional and aesthetic results.^[9,10]^

In this report, we present a case of a patient who was incidentally diagnosed with a right TMJ osteochondroma and synovial chondromatosis during a routine dental implant consultation. Most clinical studies on osteochondroma have been limited to discussions of surgical management and reconstruction within a single department. However, we planned a multidisciplinary treatment approach involving an oral and maxillofacial surgeon, an orofacial pain specialist, an oral and maxillofacial pathologist, and a dental implant clinic. We diagnosed osteochondroma based on various imaging studies, formulated a patient-specific treatment plan through a multidisciplinary approach, and successfully performed minimally invasive tumor removal. Following the surgery, we conducted postsurgical rehabilitation and provided dental implant prosthetics, resulting in satisfactory restoration of the patient’s occlusion.

## 2. Case presentation

### 2.1. Chief complaints

**Figure 1.**
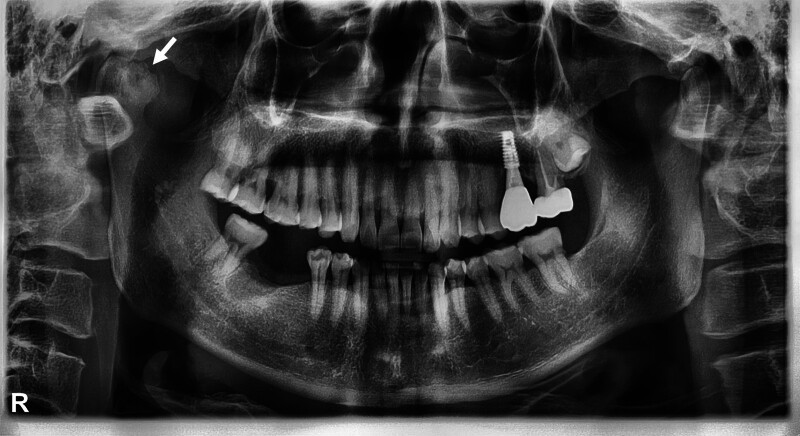
Panoramic radiograph at initial visit. The patient presented with complaints of tooth loss in the right mandibular molar region (#46, #47). An inferiorly positioned right condyle with a pedunculated, irregular mass (arrow) is visible.

A 50-year-old male presented to the dental implant clinic for a routine consultation regarding his right mandibular molars (#46, 47). During the radiographic examination, an inferiorly positioned right condyle with unusual bony irregularities was noticed (Fig. 1).

### 2.2. Medical history

The right mandibular posterior molars (#46, 47) had been extracted a year ago due to dental caries. The patient had no history of systemic diseases. Preoperative laboratory tests, however, revealed a prediabetic condition (see laboratory examinations). Upon discussion of these findings, the patient chose to pursue further testing and treatment for diabetes at a hospital in his hometown.

### 2.3. Physical examination

On physical examination, the vital signs were as follows: body temperature, 36.6 °C; blood pressure, 120/74 mm Hg; heart rate, 83 beats per minute; respiratory rate, 18 breaths per minute.

No palpable abnormalities were found in the right preauricular lesion. The maximum unassisted mouth opening (MMO), overjet (OJ), and overbite (OB) were 52 mm, 3.5 mm, and 2 mm, respectively. No shift in the midline of mandible was found.

### 2.4. Laboratory examinations

Routine blood count and coagulation tests were within normal limits. Biochemical analysis revealed a fasting glucose of 115 mg/dL and an HbA1c of 6.3%. All other values, including renal/liver function and electrolytes, were also within normal limits.

### 2.5. Imaging examinations

**Figure 2.**
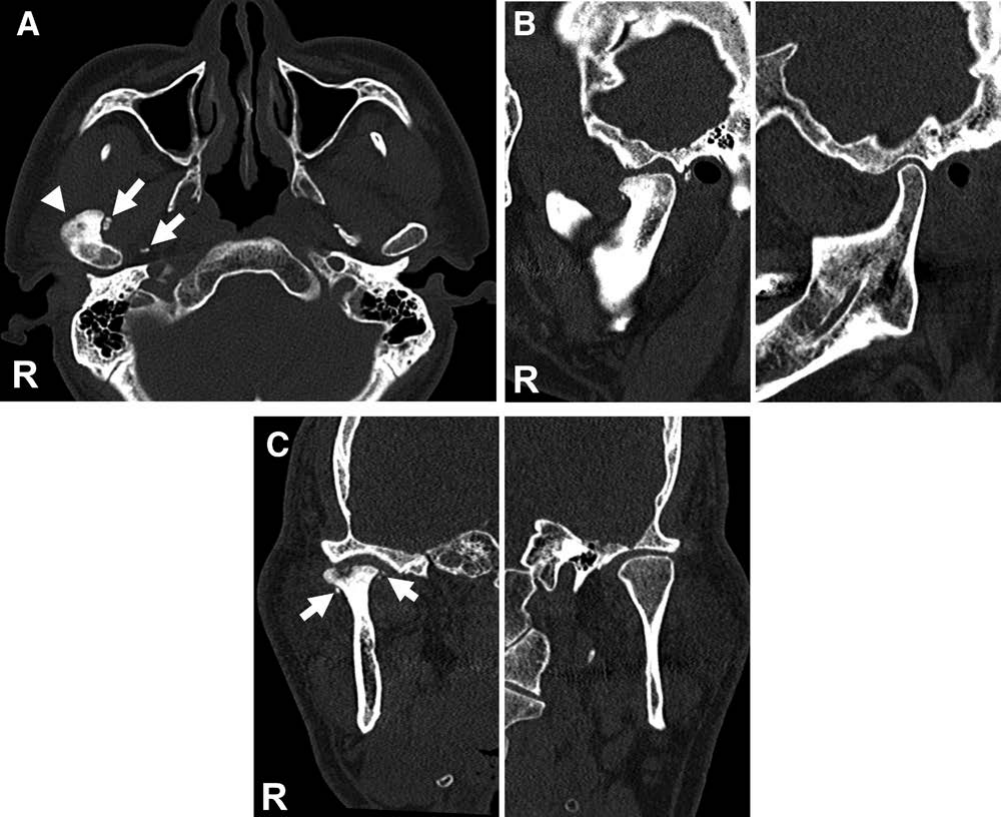
Preoperative computed tomography (CT). (A) Axial view, showing a radiopaque mass fused to anterolateral surface of the right condylar head (arrowhead) with several calcified masses (arrows). (B) Oblique sagittal view of the temporomandibular joint (TMJ), showing the right condyle displaced from the articular fossa. (C) Coronal view of the TMJ, displaying osteoarthritic changes along with several calcified fragments (arrows) on the medial and lateral aspects of the right condyle.

**Figure 3.**
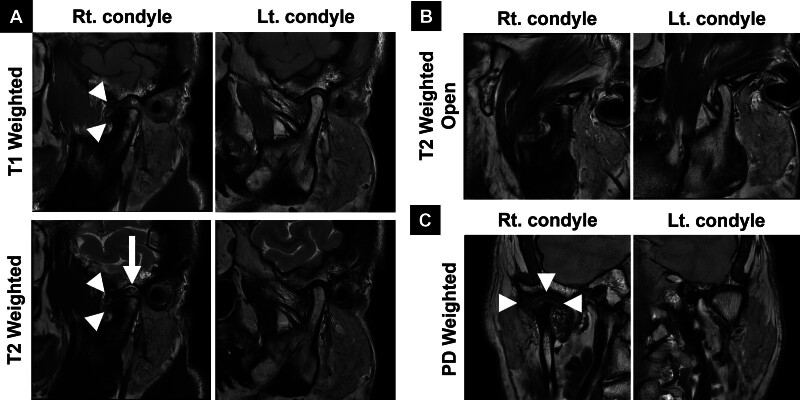
Preoperative magnetic resonance images (MRI). (A) T1 and T2-weighted oblique sagittal views in the closed-mouth position, showing the marrow and cortical continuity of osteochondroma with the underlying condyle head (arrowheads). Anteriorly displaced articular disc with joint effusion is visible in the T2 weighted images (arrows). (B) T2-weighted oblique sagittal view in the open-mouth position, showing reduction of the right articular disc, although its range of motion is slightly limited compared to the left TMJ. (C) Proton density (PD)-weighted coronal view in the closed-mouth position, showing the characteristic cartilage cap of the osteochondroma (arrowheads). TMJ = temporomandibular joint.

Considering the size and shape of the abnormality, additional imaging with computed tomography (CT) and magnetic resonance imaging (MRI) was performed. The CT scan revealed a pedunculated mass on the right condyle with multiple calcified masses (Fig. 2). The MRI showed a soft tissue mass surrounding the right condylar head (Fig. 3). Based on these findings, an initial diagnosis of osteochondroma on the right TMJ was made.

### 2.6. Multidisciplinary expert consultation

At the Dental Implant Clinic of the Armed Forces Capital Hospital, various specialties (including oral surgeons, periodontists, prosthodontists, endodontists, orthodontists, and orofacial pain specialists) work together to evaluate and collaborate on each patient’s treatment planning process.

After a multidisciplinary discussion, surgical excision was planned prior to dental implantation, due to the potential occlusal instability or discomfort following the implant prostheses. Functional rehabilitation was planned immediately after the surgery, including physical therapy, isometric jaw exercises, and an occlusal stabilization splint. The final prostheses for the dental implant were planned to be placed after achieving stable occlusion.

Informed consent was obtained from the patient prior to surgery, including consent for academic use of clinical data. This study was approved by the Institutional Review Board of the Armed Forces Capital Hospital (AFCH IRB 2024-07-001-002).

### 2.7. Surgical intervention

**Figure 4.**
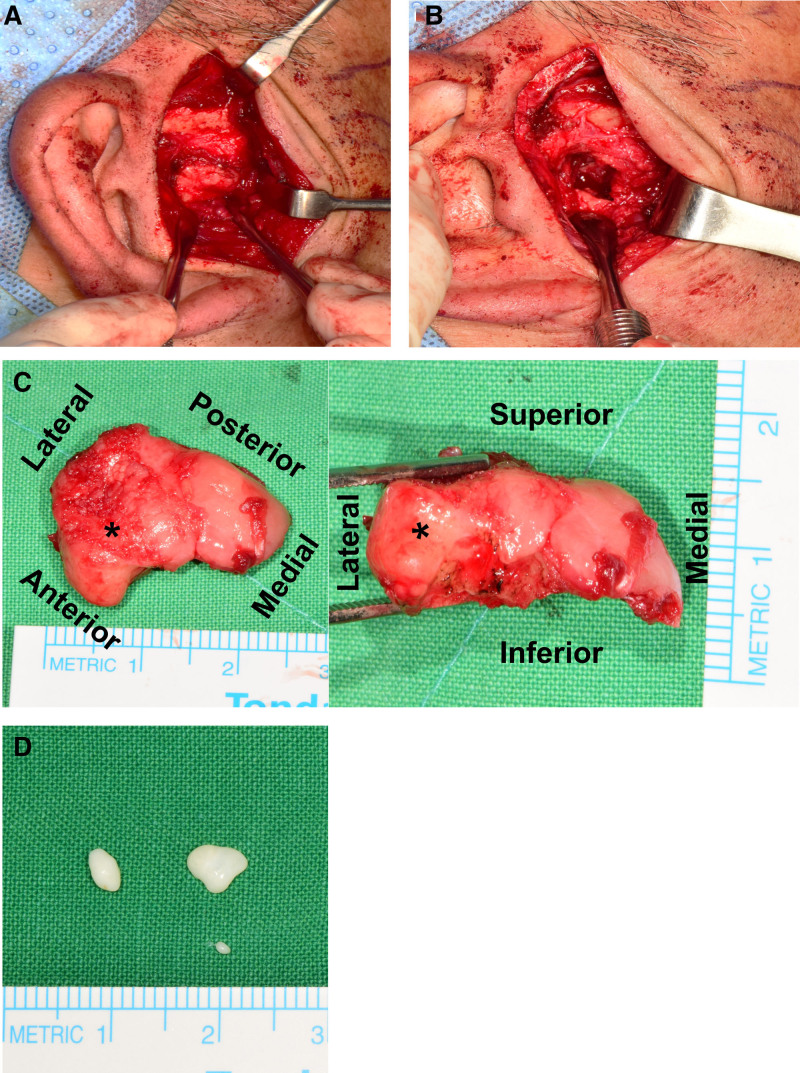
Intraoperative clinical photography. (A) Approaching the right condyle using the preauricular approach, the uneven bony mass attached to the condyle is visible. (B) A condylectomy was performed at a height of approximately 10 mm, including the tumor and condylar head. (C) The removed specimen, a lobulated bony mass (asterisk) attached to the anterolateral surface of the condylar head is visible. Its size was measured at approximately 25 mm in its largest diameter and 10 mm in height. (D) Three calcified masses around the condyle observed on the preoperative image evaluation were also identified and removed during the surgical procedure.

A low condylectomy was planned, involving excision of the mass along with 10 mm of the mandibular condyle without graft material or reconstruction. The surgical procedure was performed under general anesthesia via nasotracheal intubation, with the patient in the supine position. Standard surgical draping and aseptic techniques routinely used in oral and maxillofacial surgery were applied. Surgical access to the lesion was achieved through a preauricular approach. To minimize bleeding at the surgical site, infiltration with 2% dental lidocaine containing 1:100,000 epinephrine was administered. An incision was made through the skin and subcutaneous connective tissues using a #15 blade and electrocautery. A flap was then elevated along the level of the superficial layer of the temporalis fascia in the zygomatic arch region. Inferiorly, the dissection was continued at the same depth along the external auditory cartilage. The superficial layer of the temporalis fascia was incised at the zygomatic arch, and blunt dissection was performed inferiorly to expose the capsule of the TMJ. A vertical incision was made through the intervening tissues just anterior to the external auditory meatus, providing exposure of the TMJ capsule and zygomatic arch. After incising the capsule, the condylar head was exposed, revealing an anteriorly pedunculated, irregularly shaped tumor with an indistinct bone–tumor interface (Fig. 4). A condylectomy was then performed 10 mm inferior to the highest point of the condylar head as planned, using a surgical bur and an osteotome. Several bony nodules in the TMJ capsule were also identified and removed. No additional reconstruction techniques were employed on the resected mandibular condyle. However, an anti-adhesion agent containing hyaluronic acid (HA) (Gardix®, Hanmi Pharmacy; Seoul, South Korea) was applied to the TMJ capsule. Excised specimens were sent to the oral and maxillofacial pathologist to confirm the diagnosis. The surgical site was closed with a conventional layered suture technique, and a dressing was applied to complete the procedure.

The operation was conducted by a board-certified oral and maxillofacial surgeon with 6 years of clinical experience, including 3 years of formal surgical training and 3 years of independent specialist practice. The procedure took place at the Department of Oral and Maxillofacial Surgery, Armed Forces Capital Dental Hospital (the highest-level military dental institution in Korea). This hospital functions as a tertiary referral center within Korea’s national healthcare system, equipped with personnel and facilities comparable to those of a general academic medical center.

### 2.8. Histopathological evaluation

Histopathological examination was performed on formalin-fixed, paraffin-embedded tissue sections tissue sections stained with hematoxylin and eosin. All assessments were conducted by a board-certified oral and maxillofacial pathologist. Histological images were obtained using an Olympus BX41 microscope (Olympus Corporation, Tokyo, Japan) equipped with an eXcope X8 digital camera module (DIXI Science, Daejeon, South Korea). Image acquisition followed a standardized protocol, and representative fields were selected based on hallmark features of the lesions. Scale bars and relevant feature labels were added using Fiji (ImageJ, GitHub), an open-source image processing platform. No post-processing, such as contrast enhancement or color adjustment, was applied to the images.

**Figure 5.**
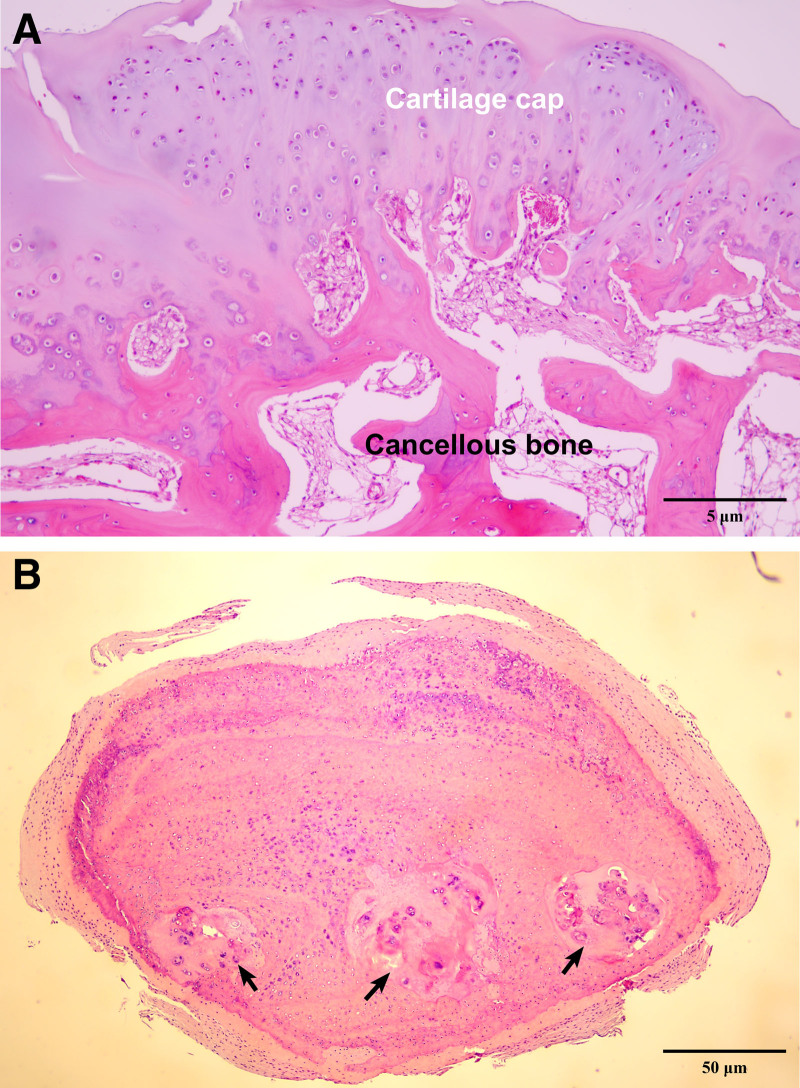
Histopathological examination of the resected specimen. (A) Photomicrograph of the resected mass of the mandibular condyle demonstrating a hyaline cartilaginous cap overlying mature cancellous bone with endochondral ossification (hematoxylin and eosin [H&E] stain; ×400 magnification). (B) Photomicrograph of synovial chondromatosis showing a nodule composed of hyaline cartilage with chondrocytes and foci of calcification (arrows) (H&E stain; ×40 magnification).

At ×400 magnification, the histological section of the mandibular condyle revealed cancellous bone capped with hyaline cartilage, with endochondral ossification between the cartilaginous cap and the underlying cancellous bone (Fig. 5A). At ×40 magnification, the calcified intra-articular masses demonstrated hyalinized cartilaginous nodules with ossification (Fig. 5B). Each finding corresponded to the typical histological features of osteochondroma and synovial chondromatosis. The synovial chondromatosis was presumed to be a secondary phenomenon associated with either osteochondroma or degenerative joint disease. Based on the histopathological evaluation, the final diagnosis was established as osteochondroma with secondary synovial chondromatosis of the right mandibular condylar head.

### 2.9. Outcome and follow-up

**Figure 6.**
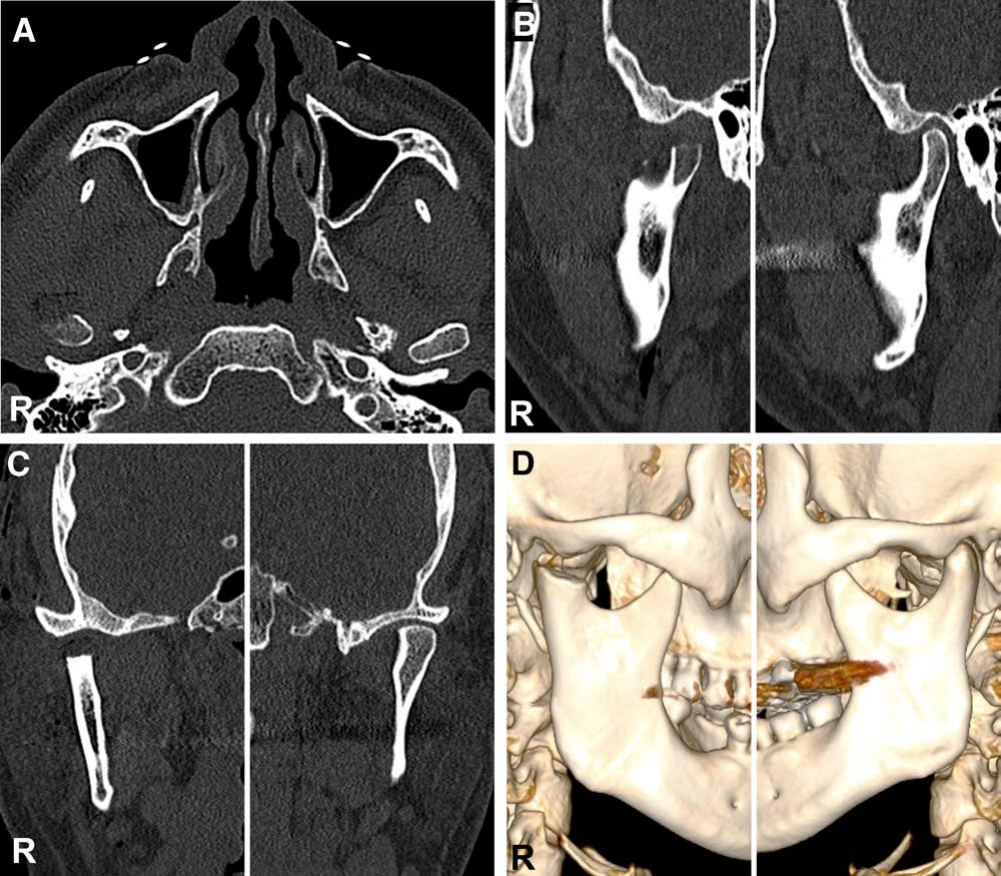
Immediate postoperative CT images. The lower part of the condylar neck was preserved. (A) Axial view. (B) Oblique sagittal view. (C) Coronal view. (D) 3-dimensional reconstruction. CT = computed tomography.

On immediate postoperative clinical examination, the patient presented with only mild hypesthesia in the preauricular region. The MMO, OJ, and OB were 46 mm, 4 mm, and 3 mm, respectively, with no midline shift. A dental implant surgery was planned for the right mandibular molar region, and an occlusal stabilization splint was fabricated (Fig. 6). The patient was instructed to wear the occlusal splint at least 8 hours a day for 3 months. Initially, the patient exhibited occlusal instability, with a 5 mm discrepancy between maximum intercuspal position (MICP) and his habitual bite. This was managed through isometric jaw exercises, physical therapy, and continuous stabilization splint treatment. The structured jaw exercise protocol, as described by Carlsson and Magnusson, is outlined in Table 1.^[11]^

**Table 1:** Structured isometric jaw exercise protocol.

Program element	Detailed description
Supervision and delivery	Supervised by an orofacial pain specialist during the inpatient phase; transitioned to a self-administered home-based program after discharge
Exercise components	- Active movements: mandibular opening, lateral excursions, and protrusion - Isometric holds with manual resistance - Passive stretching
Isometric technique	The patient applied counter-pressure using a closed fist against jaw movement (e.g., under the chin or at the jaw angle); each hold sustained for 5 seconds followed by 10 seconds of rest
Frequency and repetitions	Performed 3 times daily, with each session consisting of 10 repetitions per exercise
Program duration	Continued for at least 3 months
Exercise equipment	No external tools or specialized equipment were used
Individualization	Exercise intensity and progression were adapted to the patient’s postoperative status and tolerance
Adherence monitoring	Adherence monitored through self-reported questionnaire and follow-up interviews
Motivational strategies	Motivation reinforced by verbal encouragement and explanation of clinical benefits

One month after the surgery, the patient’s MMO was 48 mm with no change in OJ, OB, and midline shift. Mild hypesthesia was still present, but it had improved significantly, thus it was decided to continue monitoring the patient’s progress without additional treatment.

Three months after the surgery, the patient’s MMO was slightly increased to 50 mm. There were changes in occlusion; OJ and OB were 3.5 mm and 2 mm, respectively, and the midline of mandible had shifted 1 mm to the left side. The hypesthesia of the right preauricular region had completely resolved. Subsequently, a secondary implant surgery to reveal the fixture was performed.

**Figure 7.**
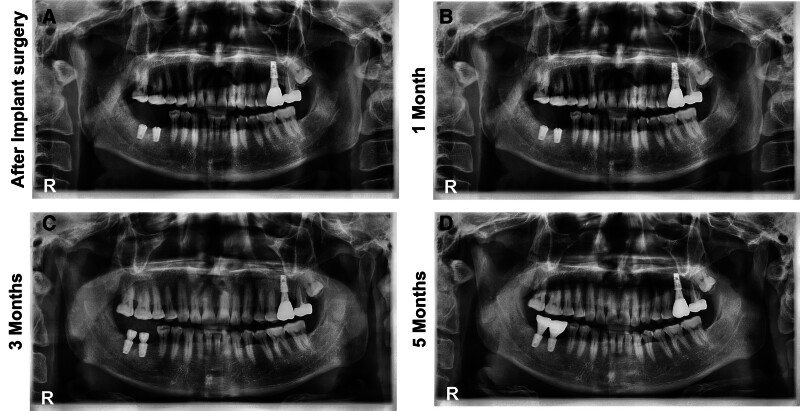
Panoramic view series following dental implant. (A) Following the removal of the osteochondroma, dental implant surgery on #46 and #47 regions with surgical extraction of #18 and #48, was performed under local anesthesia. (B) One month post-dental implantation. The patient was instructed to wear the occlusal splint at least 8 hours a day. (C) Three months post-dental implantation, a secondary implant surgery to reveal the fixture was performed and healing abutments were placed. (D) Five months post-dental implantation, the final prostheses for the right mandibular molars were delivered. CT = computed tomography.

Five months after the surgery, no clinical change in MMO, OJ, OB and the patient’s occlusion was observed. Splinted zirconia crowns on the #46, #47 implant fixtures were delivered and cemented with temporary bonding agent. The crowns were adjusted to form a uniform and consistent centric occlusion stop on both posterior regions (Fig. 7).

**Figure 8.**
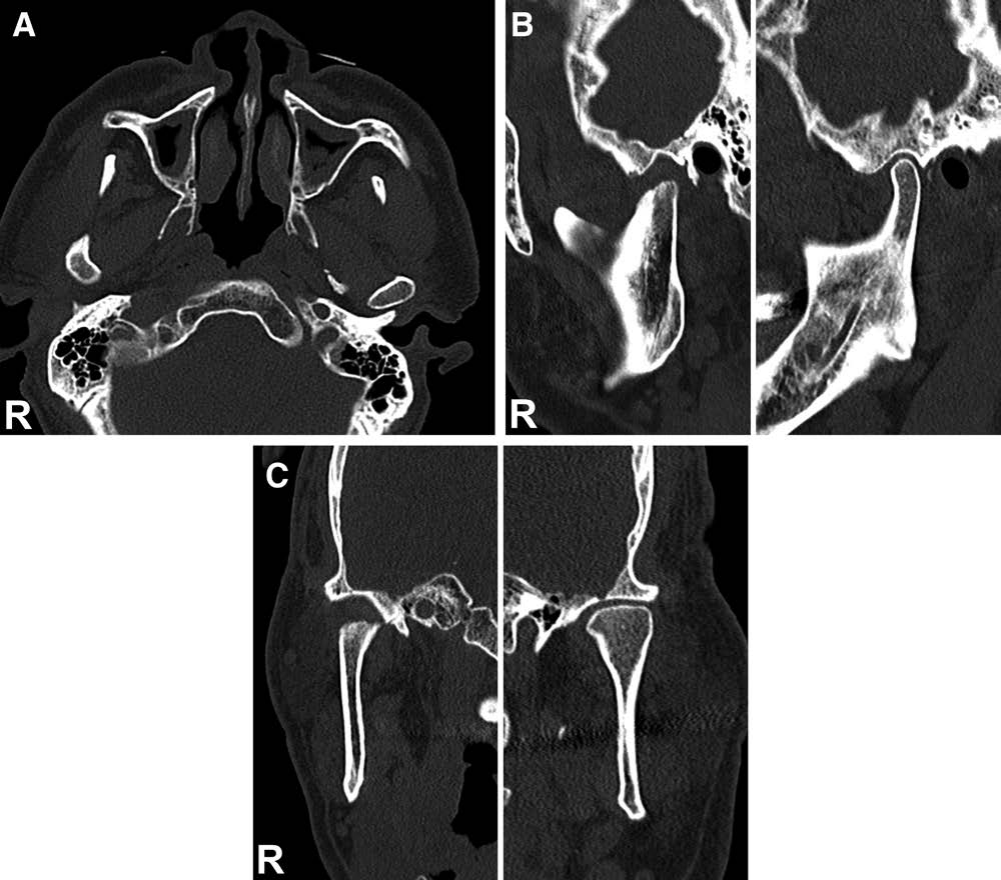
Six months postoperative follow-up CT imaging. (A) Axial view, showing some recortication of the resected area. (B) Oblique sagittal view, showing the remodeling in progress. (C) Coronal view, showing no signs of recurrence. CT = computed tomography.

**Figure 9.**
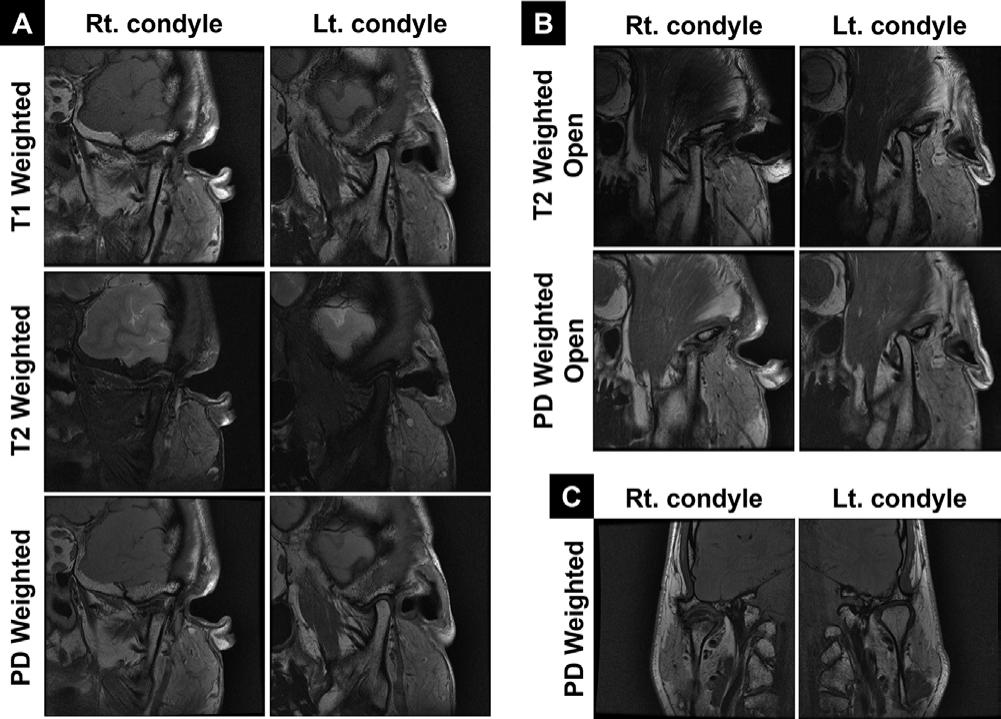
Six months postoperative follow-up MRI. (A) T1, T2, and PD-weighted oblique sagittal view in the closed-mouth position, showing resolution of the joint effusion. (B) T2 and PD-weighted oblique sagittal view in the open-mouth position, showing that the cortical bone at the upper end of the right condyle neck is not yet clear, with no signs of adhesion or ankylosis of the articular disc. (C) PD-weighted coronal view in the closed-mouth position. MRI = magnetic resonance imaging.

Six months after the surgery, the patient’s jaw position was not changed; MMO, OJ, and OB were 50 mm, 3.5 mm, and 2 mm, respectively, and the midline of mandible had shift 1mm to the left side. The patient adapted well to his new occlusion and expressed satisfaction with the rehabilitation outcome. Since the stable occlusion was achieved, the implant prostheses were cemented with a luting cement (Fujicem 2®, GC Corporation; Tokyo, Japan). Follow-up CT and MRI scans revealed remodeling of the resected mandibular condyle with no signs of recurrence or other complications (Figs. 8 and 9).

## 3. Discussion

Osteochondroma is a benign tumor characterized by a cartilage-capped bony protrusion on the external surface of a bone. Although osteochondroma in the craniofacial region is rare, it is the most common benign tumor that can arise in the TMJ.^[2,3,12]^ Multiple theories have been proposed to explain the pathophysiology of these lesions. One theory hypothesizes that stress at tendinous insertion sites, where focal accumulations of chondroprogenitor cells exist, induces the formation of these tumors.^[13]^ Additionally, some authors suggest that trauma and infection may contribute to the development of these lesions.^[14]^ In the present case, the patient denied any relevant history that may have precipitated the lesion.

Osteochondromas in the condyle generally grow gradually, with a slow manifestation of signs and symptoms. Common symptoms of TMJ osteochondromas include painless mandibular deviation, open bite, and facial asymmetry.^[3,15]^ The lesion may be asymptomatic and detected only through imaging, as in this case, underscoring the importance of comprehensive radiographic evaluations during routine dental consultations.

TMJ osteochondromas are primarily diagnosed using CT and MRI.^[15,16]^ In CT imaging, they manifest as pedunculated masses with varied density, attached either to the condyle or the coronoid process.^[16]^ These masses typically extend from the anterior or anteromedial aspect of the condyle, especially at the attachment of the lateral pterygoid muscle. This growth has the potential to cause erosion of nearby structures.^[17]^ The differential diagnoses include condylar hyperplasia, osteoma, osteoid osteoma, osteoblastoma, chondroblastoma, chondrosarcoma, giant cell tumor, and ossifying fibroma.^[17–20]^ The characteristic radiographic features of these conditions are summarized in Table 2.

**Table 2:** Radiologic differential diagnosis of mandibular condylar osteochondroma.

Diagnosis	Radiographic features
Osteochondroma	Well-defined radiopaque mass with continuity between the lesion and the underlying cortical and medullary bone, condyle enlargement presenting as a mushroom- or cauliflower-shaped exophytic mass, globular or irregular exophytic outgrowth.
Synovial chondromatosis	Multiple small radiopaque loose bodies within the joint space, joint space widening, absence of continuity with cortical bone.
Condylar hyperplasia	Enlarged condyle with preserved cortical thickness and trabecular pattern, no radiopaque mass, normal condylar shape is maintained.
Osteoma	Dense, homogeneously radiopaque lesion with smooth, well-circumscribed borders, no destructive changes, often cortical in origin.
Osteoid osteoma	Radiolucent “nidus” surrounded by dense sclerosis, not larger than 2 cm, usually contains only a single calcification.
Osteoblastoma	Well-defined expansile lesions containing small, scattered calcifications, generally > 2 cm in size.
Chondroblastoma	Well-defined radiolucent lesion with sclerotic margin, inner flocculent opacities may be present.
Chondrosarcoma	Ill-defined radiolucent lesion with mottled or “sunburst” appearance, bone destruction and cortical perforation possible.
Giant cell tumor	Multilocular radiolucent lesion with cortical breakthrough, lack a sclerotic rim, local bony destruction and soft tissue expansion.
Ossifying fibroma	Well-defined, expansile radiolucent lesion with varying degrees of central calcification, thinning of cortical plate, often displaces surrounding structures.

TMJ = temporomandibular joint.

MRI can show the boundaries between the tumor and surrounding soft tissue and hard tissue, as well as the continuity between the osteochondroma and affected bone, in a multiplanar view.^[21]^ The presence and thickness of the tumor’s cartilage cap can be directly observed as a near-fluid signal intensity structure on PD and T2-weighted MR images.^[8,22]^ Technetium-99m bone scans can also be considered as an imaging test, however, they are less specific and less commonly used for this purpose.^[12]^ In this case, CT and MR imaging modalities were instrumental in confirming the diagnosis.

Preoperative imaging revealed 3 calcified masses around the tumor, which were diagnosed as synovial chondromatosis upon postoperative pathological examination. Cases diagnosed as primary osteochondroma with secondary synovial chondromatosis, as in this study, are extremely rare.^[23]^ It is presumed that the primary osteochondroma formed initially, and as the tumor grew, it secondarily induced synovial chondrometaplasia. These loose bodies, if not sufficiently calcified, can be difficult to detect on plain X-ray and CT scans. Therefore, if secondary synovial chondromatosis is suspected, MRI can be helpful.^[23]^

The primary treatment goal for osteochondroma is the radical removal of the lesion while preserving the normal function of the TMJ and the occlusion.^[18]^ Traditionally, condylectomy followed by reconstruction surgery was preferred; however, many studies show favorable outcomes with more conservative surgical approaches.^[6,10,13,24]^ Especially for lesions confined to the condyle head, local excision or preservation-oriented condylectomy (low condylectomy), including recontouring of the condyle neck, are recommended.^[12,13,25,26]^ Total condylectomy with reconstruction is considered when both the head and neck of the condyle are involved.^[7,10,27]^

Some authors classify osteochondroma into 2 types based on the tumor’s growth patterns, each requiring different treatment approaches.^[16]^ Type 1 involves <2/3 of the condylar surface and tends to grow in a protruding expansion pattern predominantly in one direction, often treated conservatively with options such as local resection.^[16]^ Type 2 osteochondroma involves >2/3 of the condylar surface and exhibits a globular expansion pattern in multiple directions, typically necessitating condylectomy and reconstruction surgery.^[16,27]^

Conservative treatment carries the risk of inadequate tumor removal, potentially leading to tumor recurrence or malignant transformation.^[8]^ However, the overall recurrence rate of typical osteochondromas is <2%, and recurrence in the mandible is rarely reported.^[7]^ Therefore, thorough preoperative assessment and rational treatment planning can achieve favorable clinical outcomes with conservative approaches.

The preauricular approach is the most common method for TMJ tumor excisions but carries the risk of facial scarring and auriculotemporal nerve damage.^[28]^ To minimize this complication during preauricular approaches, it is advisable to dissect as close as possible to the external auditory canal. Sensory loss in the preauricular area due to nerve damage typically resolves without treatment. In the present case, the patient initially had paraesthesia in the preauricular region but resolved spontaneously within 3 months.

HA is a crucial component of synovial fluid, reducing joint surface friction and maintaining tissue elasticity.^[29]^ Injection of HA may improve jaw function and relieve pain in the TMJ.^[30,31]^ In this case, HA-containing anti-adhesive agents were injected into the condyle capsule to prevent tissue adhesions and alleviate pain. The patient reported minimal postoperative pain and discomfort during mouth-opening exercises. This allowed for the implant surgery, initially planned for outpatient care after discharge, to be performed during the admission period.

In implant dentistry, a multidisciplinary approach is essential for planning treatment and achieving optimal outcomes.^[32]^ Recently, the integration of advanced digital dental technologies (such as intraoral scanners and 3D printing) with multidisciplinary planning has been shown to enable more precise patient-specific surgical strategies and minimally invasive procedures with favorable clinical outcomes.^[33]^

Although this case did not employ the latest digital dental technologies, advanced imaging with CT and MRI enabled precise diagnosis and treatment planning through close collaboration with multiple dental specialists. Unlike recent reports focused solely on minimally invasive surgery,^[25,26]^ this case also describes postoperative functional rehabilitation and occlusal reconstruction led by an orofacial pain specialist, underscoring the integrated roles of different specialties in managing osteochondroma of the mandibular condyle.

Postsurgical rehabilitation, including an occlusal stabilization splint, isometric jaw exercises and physical therapies, is crucial for restoring occlusion and jaw function. Occlusal stabilization splints offer a new, optimal jaw position for patients who have undergone TMJ surgeries, helping them adapt the jaw to its new position after surgery.^[34]^ Additionally, stabilization splint treatment has shown effectiveness in eliminating centric relation-maximum intercuspation discrepancies.^[35]^ Although the exact mechanism is yet to be discovered, it is believed that relieving the hyperactivation of the jaw elevator muscles might play a role.^[35]^

Clinical success in correction of iatrogenic occlusal instability using isometric jaw exercises has been described.^[36]^ Isometric jaw exercises relax and strengthen the muscles of mastication and help the closing muscles guide the dentition into MICP. Physical therapies, including ultrasound therapy and transcutaneous electrical nerve stimulation, were used for the patient. Ultrasound therapy generates heat in deep tissues, increasing blood flow, reducing postoperative pain, and promoting bone healing.^[37]^ Transcutaneous electrical nerve stimulation is primarily used for sensory counter-stimulation to modulate pain. However, when used with varied frequency and intensity, it can also aid muscle relaxation.^[38]^ These interventions addressed the initial occlusal instability and helped to gradually correct the 5 mm discrepancy between the MICP and the habitual bite, leading to a stable and functional occlusion. The patient’s adherence to the rehabilitation regimen and the combined expertise of the healthcare team were crucial in achieving a favorable outcome.

Long-term follow-up is essential for patients who have undergone surgical excision of TMJ osteochondromas to monitor for potential recurrence and to ensure the continued success of the rehabilitation efforts. The patient’s positive adaptation and satisfaction with the final prosthesis further highlight the effectiveness of a collaborative treatment approach.

## 4. Conclusion

In conclusion, the multidisciplinary management of TMJ osteochondromas, as demonstrated in the present case, is essential for achieving favorable outcomes. Evidence-based recommendations include using comprehensive diagnostic imaging to accurately assess the lesion, followed by a conservative surgical approach tailored to the specific characteristics of the lesion. The application of coordinated postoperative care, such as physical therapy and occlusal stabilization, supports functional rehabilitation and enhances patient satisfaction. Clinicians can incorporate these strategies into their clinical practice to improve patient outcomes in similar cases. Future research should focus on refining conservative surgical techniques and exploring the long-term efficacy of various rehabilitation protocols.

## Acknowledgments

The authors thank Prof Jung-Hoon Yoon, for his contribution to the study.

## Author contributions

**Conceptualization:** Hyun Nam, Kyung Su Shin.

**Formal analysis:** Hyun Nam, Da Eun Ko.

**Visualization:** Da Eun Ko, Kyung Su Shin.

**Writing – original draft:** Hyun Nam, Kyung Su Shin.

**Writing – review & editing:** Hyun Nam, Da Eun Ko, Kyung Su Shin.
